# Volatile Profile in Yogurt Obtained from Saanen Goats Fed with Olive Leaves

**DOI:** 10.3390/molecules25102311

**Published:** 2020-05-14

**Authors:** Francesca Bennato, Denise Innosa, Andrea Ianni, Camillo Martino, Lisa Grotta, Giuseppe Martino

**Affiliations:** 1 Faculty of BioScience and Technology for Food, Agriculture and Environment, University of Teramo, Via Renato Balzarini 1, 64100 Teramo, Italy; fbennato@unite.it (F.B.); dinnosa@unite.it (D.I.); aianni@unite.it (A.I.); lgrotta@unite.it (L.G.); 2 Istituto Zooprofilattico Sperimentale dell’Abruzzo e del Molise “G. Caporale”, Via Campo Boario 37, 64100 Teramo, Italy; c.martino@izs.it

**Keywords:** goats’ milk yogurt, Saanen goat, olive leaves, unsaturated fatty acid, volatile compound, free fatty acid, aldehyde

## Abstract

The aim of this study was to evaluate the development of volatile compounds in yogurt samples obtained from goats fed a dietary supplementation with olive leaves (OL). For this purpose, thirty Saanen goats were divided into two homogeneous groups of 15 goats each: a control group that received a standard diet (CG) and an experimental group whose diet was supplemented with olive leaves (OLG). The trial lasted 28 days, at the end of which the milk of each group was collected and used for yogurt production. Immediately after production, and after 7 days of storage at 4 °C in the absence of light, the yogurt samples were characterized in terms of fatty acid profile, oxidative stability and volatile compounds by the solid-phase microextraction (SPME)–GC/MS technique. Dietary OL supplementation positively affected the fatty acid composition, inducing a significant increase in the relative proportion of unsaturated fatty acids, mainly oleic acid (C18:1 *cis*9) and linolenic acid (C18:3). With regard to the volatile profile, both in fresh and yogurt samples stored for 7 days, the OL supplementation induced an increase in free fatty acids, probably due to an increase in lipolysis carried out by microbial and endogenous milk enzymes. Specifically, the largest variations were found for C6, C7, C8 and C10 free fatty acids. In the same samples, a significant decrease in aldehydes, mainly heptanal and nonanal, was also detected, supporting—at least in part—an improvement in the oxidative stability. Moreover, alcohols, esters and ketones appeared lower in OLG samples, while no significant variations were observed for lactones. These findings suggest the positive role of dietary OL supplementation in the production of goats’ milk yogurt, with characteristics potentially indicative of an improvement in nutritional properties and flavor.

## 1. Introduction

The flavor development in dairy products is an important factor determining its acceptability and preference and is strongly affected by the combination of a wide range of compounds, mostly produced by a series of biochemical events involving the metabolism of residual lactose, lactate, and citrate, lipolysis and proteolysis. Among the mentioned catabolic processes, lipolysis is undoubtedly the mechanism that, more than others, contributes to the release of flavor-affecting compounds, mainly free fatty acids (FFAs), aldehydes, alcohols, esters, methyl ketones, γ- and δ-lactones. Milk and cheese are, in fact, characterized by relevant levels of short- and medium-chain fatty acids, whose specific composition is strongly influenced by the animal species and the administered dietary treatment [1].

In recent years, an increase in consumer demand for goats’ milk and its derivatives, due to the high nutritional features and health-promoting benefits of these products, has been observed. Goat dairy products were, in fact, reported to be characterized by proteins with lower allergenicity in comparison to bovine products, and higher concentrations of bioactive compounds, attributed to their greater digestibility [2,3]. Specifically, a growing interest has been developed towards yogurt, commonly considered a safe and nutritious food and not only being rich in vitamins and minerals, but also in calcium and proteins [4]. Furthermore, it was demonstrated that yogurt consumption is helpful for consumers affected by specific gastrointestinal conditions, mainly those associated with lactose intolerance and constipation [5].

In the past, several studies were conducted on the inclusion of different natural sources of bioactive compounds in goats’ milk yogurt in order both to improve nutritional value and reduce the unpleasant “goaty” aroma and aftertaste, commonly associated with decreased acceptance by consumers [6,7]. In time, studies performed in the sector of ruminant husbandry led to the development of feeding strategies able to influence the chemical composition of animal products. In this regard, several experiments have been performed by supplementing animals’ diets with plant matrices, especially agro-industrial byproducts rich in bioactive compounds with interesting functions from a biochemical point of view. These strategies have shown positive effects on several fronts, preserving, in many cases, animal welfare [8], improving the nutritional quality of animal products [9,10], and inducing, especially in dairy products, the development of volatile compounds capable of influencing the aroma and taste [11].

Byproducts derived from olive oil production are accumulated yearly in high amounts in the Mediterranean area, and their disposal represents an issue of great importance both from an environmental and economic point of view. Several studies focused their attention on the valorization of these byproducts as feeding supplements for farm animals, both ruminant and monogastric [12,13]. Specifically, olive leaves (OL) have been reported to be a cheap raw material that can be used as a source of phenolic bioactive compounds with antioxidant, antihypertensive and anti-inflammatory functions, such as oleuropein, hydroxytyrosol, verbascoside, apigenin-7-glucoside and luteolin-7-glucoside [14].

Considering the biological relevance of this byproduct, the purpose of this work is to evaluate the effects of dietary OL supplementation of lactating dairy goats on the development of volatile compounds in fresh and stored yogurt samples, with specific attention paid to compounds derived from lipolytic events.

## 2. Results

### 2.1. Chemical Properties of Yogurt Samples

The evaluation of chemical properties in yogurt samples did not evidence significant variations between the control group that received a standard diet (CG) and the experimental group whose diet was supplemented with olive leaves (OLG) in relation to moisture (85.74 ± 0.26% vs. 85.17 ± 0.49% in CG and OLG respectively, *p* > 0.05) and the amount of total lipids (19.81 ± 1.22% vs. 19.68 ± 1.35% in CG and OLG respectively, *p* > 0.05). The only significant difference was observed for pH values that decreased in yogurt obtained from goats fed the dietary OL supplementation (4.69 ± 0.02 vs. 4.43 ± 0.04 in CG and OLG samples respectively, *p* < 0.05).

### 2.2. Fatty Acid Composition

The dietary OL supplementation in the goats’ diet induced several modifications in the fatty acid composition of yogurt samples. As reported in Table 1, in OLG samples, we observed a significant increase in the relative proportion of total monounsaturated fatty acids (MUFA; 22.25 ± 0.92% vs. 24.80 ± 0.77% for CG and OLG respectively, *p* < 0.05) and total polyunsaturated fatty acids (PUFA; 4.62 ± 0.14% and 4.84 ± 0.07% for CG and OLG respectively, *p* < 0.05). With specific regard to the sum of the saturated fatty acids (SFA), a slight decrease was instead observed in OLG samples, although this variation was not significant (*p* > 0.05).

With reference to individual fatty acids, the OLG yogurt samples were characterized by a decrease in the relative proportion of lauric acid (C12:0, *p* < 0.05), myristic acid (C14:0, *p* < 0.05), palmitic acid (C16:0, *p* < 0.05), and linoleic acid (C18:2, *p* < 0.05), while a significant increase in the relative proportion was observed for stearic acid (C18:0, *p* < 0.01), oleic acid (C18:1 *cis*-9, *p* < 0.01), linolenic acid (C18:3, *p* < 0.01), and conjugated linoleic acids (CLA, *p* < 0.05). In addition to this, in OLG samples, the ratio MUFA/SFA and UFA/SFA was significantly higher (*p* < 0.05), whereas no variations were evidenced in the individual desaturation indices calculated for C14:1, C16:1, C18:1 and CLA.

### 2.3. Evaluation of the Oxidative Stability in Fresh and Stored Yogurt

The evaluation of the overall antioxidant potential in fresh yogurt highlighted a higher antioxidant capacity in OLG samples (79.69 ± 4.76 µmol TEAC/g and 89.77 ± 5.03 µmol TEAC/g for CG and OLG respectively, *p* < 0.05).

**Figure 1:**
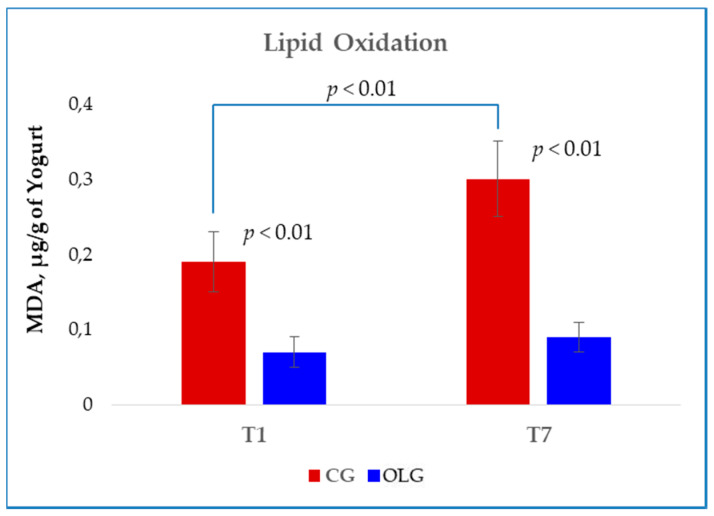
Lipid oxidation evaluated in yogurt samples obtained from goats fed the standard diet (CG) and goats that received the dietary supplementation with olive leaves (OLG). Samples were analyzed after 1 day (T1) and 7 days of storage (T7) at 4 °C. Malondialdehyde (MDA).

The oxidative process on the lipid component was instead determined by a Thiobarbituric Acid Reactive Species (TBARS) test, both after 1 day (T1) and 7 days (T7) of storage at +4 °C and the results are shown in Figure 1. In T1 samples, the amount of malondialdehyde (MDA) was lower in OLG samples compared with CG samples (*p* < 0.01). In T7 samples, the same trend was observed, although a marked increase in the difference between CG and OLG samples was found (*p* < 0.01). By comparing the obtained results on the basis of the storage time (T1 vs. T7), a significant increase in oxidation was evidenced in CG yogurt (*p* < 0.01), while no variations were induced by the OL dietary enrichment.

### 2.4. Identification of Volatile Compounds

**Figure 2:**
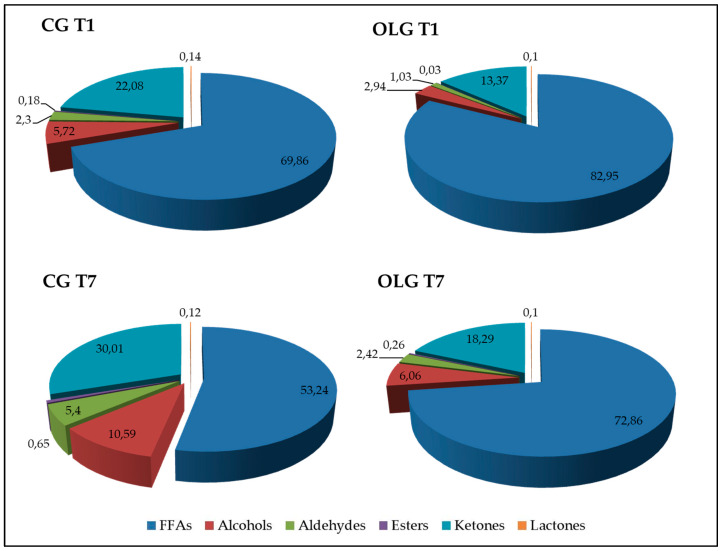
Schematic representation of relative proportions (%) of volatile compound families detected in yogurt samples obtained from goats fed the standard diet (CG) and goat that received the dietary supplementation with olive leaves (OLG). Samples were analyzed after 1 day (T1) and 7 days of storage (T7) at 4 °C. Free fatty acids (FFAs).

In all samples, we found 26 volatile compounds (VOCs): seven free fatty acids (FFAs), five alcohols, five aldehydes, one ester, six ketones and two lactones. As schematized in Figure 2, both after 1 and 7 days of storage, the dietary OL supplementation was effective in inducing an increase in the relative proportion of FFAs (*p* < 0.05 and *p* < 0.01, for T1 and T7 respectively) and a significant decrease in the relative proportion of alcohols (*p* < 0.05 and *p* < 0.01, for T1 and T7 respectively), aldehydes (*p* < 0.01), esters (*p* < 0.05), and ketones (*p* < 0.05).

Table 2 reports the specific compositions of the listed VOC families. After 1 day after yogurt production, all the alcohols found were lower in OLG samples in comparison with CG samples: 1-hexanol (*p* < 0.01), 2-ethyl-hexan-1-ol, (*p* < 0.05), 1-heptanol (*p* < 0.001), 1-octanol (*p* < 0.01), and 1-nonanol (*p* < 0.001). After 7 days of storage, such a condition persisted, with the only exception being 1-hexanol, 2-ethyl, for which no significant variations were evidenced. With regard to the aldehydes, only nonanal was significantly lower in T1 samples derived from the dietary OL supplementation (*p* < 0.01), while, at T7, this behavior was also observed for heptanal (*p* < 0.05). Among the FFAs, the OLG samples stored for 1 day after the yogurt production were characterized by higher relative proportions of octanoic acid (*p* < 0.05) and decanoic acid (*p* < 0.05). The finding concerning the octanoic acid was also confirmed after 7 days of yogurt storage (*p* < 0.01) and, in addition to this, the same samples also showed a decrease in the relative proportions of hexanoic acid (*p* < 0.05) and heptanoic acid (*p* < 0.01). Concerning ketones, both after 1 and 7 days of storage, we observed lower relative proportions for 2-heptanone (*p* < 0.05) and 2-nonanone (*p* < 0.01). Butyl heptanoate was the only ester to be detected and was less represented in OLG yogurt compared to CG, both at T1 and T7 (*p* < 0.01). Finally, no significant variations were evidenced for the two identified lactones: δ-decalactone and δ-dodecalactone (*p* > 0.05).

## 3. Discussion

Flavor is an important factor determining the acceptability of food products. With specific regard to dairy products, the sensory properties are largely dependent on the development of volatile flavor compounds mainly derived from biochemical processes that degrade fat and proteins present in milk. Considerable knowledge has been accumulated on the wide range of aromatic compounds contributing to the development of flavor in yogurt. Most of these compounds include VOCs already present in milk and compounds derived from fermentation processes [15]. Dietary OL supplementation was able to influence VOC development in goats’ milk yogurt, with variations particularly evident in the production of lipolytic catabolites.

The experimental feeding strategy was, first of all, able to induce significant changes in the fatty acid profile of yogurt samples. The relationship established between fat and flavor development in dairy products is complex; however, it should be considered that fat is a rich reservoir of flavors, as a consequence of the tendency of many aromatic compounds to be soluble in fat rather than in water [16]. Furthermore, the proportion and structure of fat may affect the rheology of the product, and thus its dispersion in the mouth. It is also well known that lipids present in food represent precursors of several flavor compounds, since may undergo spontaneous oxidation or enzymatic hydrolysis, which are essential for flavor development [17]. It is therefore plausible that variations at the substrate level could have influenced the extent of these biochemical mechanisms, thus influencing VOC production. The most relevant variation was associated to the increase in the relative proportion of MUFAs and PUFAs in OLG samples. Since the calculated desaturation indices (for C14:1, C16:1, C18:1 *cis*-9 and CLA) did not show significant differences, it is presumable that the changes observed in the fatty acid composition are totally ascribable to the different diets administered to goats. Variations found in the calculation of these desaturation indices are, in fact, generally attributed to a greater expression or activity of stearoyl-CoA desaturase (SCD), also known as Δ^9^-desaturase [18]. This enzyme plays a leading role in the lipid metabolism of the mammary gland, due to its ability to catalyze the addition of a double bond in the cis-Δ^9^-position in a large spectrum of medium- and long-chain fatty acids [19]. The substrates with which this enzyme interacts with greater affinity are precisely the acyl-CoA of C14, C16, C18 and trans-11 C18:1, which are respectively converted into C14:1, C16:1, C18:1 cis-9, and C18:2 *cis*-9 *trans*-11 [20].

The presence in the OLG samples of lower relative proportions of SFA lends itself to be discussed from different points of view. First of all, the dietary intake of SFA for humans is notoriously associated with an increased risk of developing cardiovascular diseases [21], and the importance of limiting the concentration of these compounds in foods acquires particular relevance in ruminant products because of the biohydrogenation mechanisms that are responsible for dietary PUFA conversion into SFA or MUFA [22]. Additionally, it must be specified that the improvement in the health indices of a food product is more specifically associated with the increase in omega-3 fatty acids and, over time, different feeding strategies have been tested in the zootechnical field, with the aim of obtaining the enrichment of such compounds in goats’ milk and its derived dairy products [23]. Therefore, in this study, the increase in the relative proportion of linolenic acid (C18:3) observed in yogurt samples obtained from goat fed the OL supplementation acquires particular value. This finding is consistent with what was recently evidenced in a similar study focused on the evaluation of nutritional quality of Ricotta cheese made from goats’ milk [24], and can be fully justified by the fact that C18:3 has been reported to be the major fatty acid in OL [25].

In addition to what has been reported, it must be considered that the increase in the relative proportion of unsaturated forms exposes these food products to greater susceptibility towards oxidative processes, which take place in most cases as an effect of the action of reactive species that are able to interact with C=C double bonds, producing peroxides [26]. The PUFA tendency to undergo oxidation is an aspect of remarkable importance for the food industry, since high concentrations of these compounds can induce detrimental effects on food nutritional quality and may represent a cause of concern for food safety [27]. Despite what has been reported, it should be said that, in the present study, the antioxidant potential in OLG yogurt samples was higher than in the CG samples—a finding also consistent with the results obtained by the TBARS test, which showed a greater resistance to lipid peroxidation in yogurt obtained from goats fed the dietary OL supplementation. The enrichment of ruminants’ diets with plant matrices rich in compounds of high value from a biological point of view is generally associated with an improvement in the oxidative stability of animal productions [28,29]. Benavente-Garcia et al. [30] performed an accurate qualitative and quantitative characterization of the phenolic compounds present in OL extracts, assigning each compound its own antioxidant potential. Oleuropein was indicated as the most represented phenolic compound in the extracts. However, the greatest antioxidant function was attributed to hydroxytyrosol which is derived from the oleuropein hydrolysis. There is not much information on the bioavailability of these compounds in ruminants and, above all, there is a lack of information on their possible transfer to milk in the form of secondary metabolites able to act as antioxidants. However, even other studies conducted in the past on dairy cows and goats have highlighted the better oxidative stability of dairy products obtained by feeding animals with olive oil byproducts [13,24].

With regard to the volatile profile, numerous studies have been conducted in recent years, which highlight the diet’s role in influencing the production of volatile flavor compounds in ruminant products [11]. Most of the compounds identified in all samples belong to the FFA group, which is testament to the prevalence of lipolytic processes compared to other catabolic mechanisms. Both in the freshly produced yogurt (T1) and in the one kept at 4 °C for 7 days (T7), carboxylic acids are present in higher relative proportions than in the OLG samples. In T1, this finding is mainly due to the greater presence of octanoic and decanoic acids. Both of these compounds have been reported to contribute to the characteristic animal-like, rancid and “soapy” flavor notes [31]. At T7, the data regarding octanoic acid is confirmed, but it is nevertheless interesting to note the slight but significant reduction in the relative proportion of hexanoic and heptanoic acids. These compounds are associated with pungent, rancid and flowery notes [32]; however, their relative concentrations place them in second place with respect to octanoic acid as contributors to the overall flavor. Apart from the question concerning the flavor development, the greater presence of octanoic acid in OLG samples also lends to be discussed from the perspective of food safety. In fact, the study conducted by Kinderlerer and Lund [33] reported the ability of this compound to inhibit the growth of 10 strains of *Listeria monocytogenes* and two strains of *L. innocua*, with a minimum inhibitory concentration (MIC), which was comparable with that determined in several cheeses. In particular, in T7 samples, the dietary OL supplementation induced significant differences between volatile short-chain fatty acids, while no significant changes were found for total long-chain fatty acids (nonanoic and decanoic). Since no variations were detected in the total lipid content between CG and OLG, it is plausible that this data is the result of the differential expression or activity of the bacterial lipases, whose reaction kinetics could have been influenced by secondary OL metabolites, which reached the milk through the mammary gland. However, in this study, we did not characterize of the OL metabolites in milk; therefore, further and more specific assessments are needed to clarify this aspect.

FFAs also contribute to the formation of cheese flavor indirectly, since they represent the precursors of other chemical families: methyl ketones, secondary alcohols, esters, aldehydes and lactones [34]. In this study, the second group of VOCs in order of importance was that of methyl ketones, which are formed following the oxidation of FFAs to β-ketoacids and subsequent decarboxylation to corresponding methyl ketones [16]. These compounds are reported to be the major determinants for the characteristic flavor of blue-veined and surface-mold-ripened cheeses, as a consequence of their typical odors and low perception thresholds [35]. However, in yogurt, many of these compounds have also been associated with well-defined aromatic notes. In this study, a specific pattern has been observed with regard to 2-heptanone and 2-nonanone, to which the ability to confer floral and green-fruity notes is attributed [15]. These compounds were found to be predominant in several dairy products, and their concentrations are reported to increase during the early stage of ripening, after which levels undergo reduction [36]. This behavior is generally observed over a period of a few weeks, and since the yogurt samples considered in the present study have been stored for few days, presumably only the initial part of the phenomenon, characterized by the increase in the concentrations of these compounds, has been highlighted. The peculiarity concerns the fact that both in T1 and T7 samples, the dietary OL supplementation seems to negatively influence the production of methyl ketones. The most plausible explanation could be precisely the greater antioxidant potential that should characterize the OLG samples and which would have protected the FFAs from oxidation to β-ketoacids.

In the presence of free radicals, the unsaturated fatty acids characterizing the matrix of dairy products can undergo non-enzymatic intrachain oxidation, giving origin to hydroperoxides, which rapidly decompose to form compounds such as propanal, hexanal, heptanal, octanal, nonanal or unsaturated aldehydes, whose presence is commonly associated with a “green grass-like” aroma [16,37]. The accumulation of aldehydes as a consequence of lipid oxidation is responsible for the off-flavor development during yogurt storage. For this reason, Carrillo-Carrion et al. [38] proposed to use the total concentration of volatile aldehydes (mainly from C5 to C9) as a marker of yogurt deterioration during storage. The dietary OLG supplementation was effective in curbing such mechanisms, especially after 7 days of storage, and this evidence also correlates with a presumed improvement in the yogurt’s oxidative stability. This finding is consistent with what has been already observed in other dairy products obtained from ruminants fed diets supplemented with vegetable matrices rich in bioactive compounds, mainly polyphenols [11]. In addition to this, it should be mentioned that the oxidation of unsaturated fatty acids can even be mediated by microbial lypoxygenases. These enzymes are described as non-heme iron enzymes able to catalyze the dioxygenation of PUFAs to hydroperoxy fatty acids. Although limited information is available on the bacterial isoforms of these enzymes, it is, however, known that the preferred substrate is represented by linoleic acid [39]. Therefore, the higher concentration of linoleic acid found in the CG yogurt samples could, in part, have favored a greater accumulation of aldehydes; however, it should also be considered that the extent of this enzymatic phenomenon should have been limited in any case, given the relative percentage of linoleic acid compared to other fatty acids.

Aldehydes that accumulate in dairy products during storage or ripening may be reduced into the corresponding primary alcohols, which are considered transitory compounds [16]. Both in T1 and T7 samples, the dietary OL intake led to a limitation of this biochemical process, resulting in lower relative proportions of all the identified alcohols, precisely 1-hexanol, 1-heptanol, 1-octanol, 1-nonanol and 2-ethyl-hexan-1-ol. Contrary to what was expected, in all samples, the presence of ethanol, which is considered one of the most characteristically volatile compounds participating in the formation of yogurt flavor, was not evidenced. Ethanol can be alternatively released during yogurt manufacture by the starter lactic acid bacteria (LAB), through a mechanism that involves the conversion of lactose into lactate with consequent pH lowering, and lactate metabolization into formate, acetaldehyde and ethanol [15,34]. Therefore, in this case, any secondary metabolites derived from bioactive compounds consumed by the goats through the OL intake could have already influenced the metabolism of the microbial forms involved in the yogurt production in the early stages of the procedure.

Alcohols that accumulate in yogurt can also react with free acids to produce esters such as ethyl-acetate and butyl-acetate. In all the analyzed samples, the only identified ester was butyl heptanoate, which showed significantly lower concentrations in the OLG yogurt. This is a compound not commonly found in yogurt, whose synthesis mechanisms and specific contributions to the development of flavor require further and more specific evaluations.

## 4. Materials and Methods

### 4.1. Experimental Design, Yogurt Manufacturing Protocol and Sampling

Thirty Saanen goats, homogeneous in age, lactation period, and number of births were used for the experiment. The trial lasted 28 days, in which the nutritional needs of goats in lactation were satisfied. Animals were randomly divided into two groups, a control group (CG) and an experimental group (EG) that received the dietary enrichment of 350 g/die/goat of olive leaves.

Regarding the yogurt production, whole raw goats’ milk was pasteurized at 92 °C. Then, the milk was cooled at 40 °C, inoculated with a lactic starter mixture (*Streptococcus thermophilus* and *Lactobacillus delbrueckii* subsp. *Bulgaricus*; Santamaria Srl, Burago di Molgora (MB), Italy), portioned into 300-mL glass containers and incubated at 45 °C for 12 h. For each group of goats, 12 yogurts were produced; six yogurts for each treatment were sampled one day after production (T1) and the remaining six for each group were sampled after seven days (T7) of storage at 4 °C. All the samples were collected and maintained at −20 °C until the analysis.

### 4.2. Chemical Analysis

The evaluation of the chemical properties of yogurt samples were performed according to AOAC methods [40]. Briefly, pH was measured at 25 °C by using a pH-meter HD 8705 (Delta OHM, Caselle di Selvazzano (PD), Italy) and moisture content (method 933.05) was determined on 5 g of sample left in the stove for 6 h at 105 °C.

With regard to the evaluation of total lipids, 3 g of yogurt was treated with 5 mL of ethanol (Sigma Aldrich, Milan, Italy) and 750 µL of ammonium hydroxide 25% in water to obtain protein precipitation. Then, three consecutive extractions with diethyl ether and petroleum ether (Sigma Aldrich, Milan, Italy) were performed and, every time, the supernatant was recovered in a previously calibrated flask. At the end of the extraction, a rotary evaporator was used to remove the solvent, and the flask containing the lipids was then moved into the stove at 40 °C for 20 min to eliminate humidity. After cooling at room temperature in a dryer, all flasks were weighed to calculate the total fat percentage for each sample. Results were expressed on a dry matter (DM) basis.

### 4.3. Fatty Acid Composition

The evaluation of the fatty acids (FA) profile was performed as previously described with slight modifications [41]. Briefly, 60 mg of total fat was solubilized in 1 mL of hexane and methylated with 500 µL of sodium methoxide 2 N in water to obtain the fatty acid methyl esters (FAMEs). Then, 1 µL of methylated extract was injected in the gas chromatograph (GC; ThermoScientific, Waltham, MA, USA) coupled with a flame ionization detector (FID) and equipped with a column Restek Rt-2560 (100 m, 0.25 mm ID, 0.20 µm df). The injector and detector temperature were both set at 280 °C and hydrogen was used as carrier gas at a flow rate of 1 mL/min. The chromatographic run lasted 56 min and the oven temperature was first held at 80 °C for 10 min, then increased from 80 °C to 172 °C at 4 °C/min and held for 30 min, and finally increased from 172 °C to 190 °C at 4 °C/min and held for 10 min. The identification of each FAME was performed by comparing the peak retention time with those obtained from a mix of analytical standards (F.A.M.E. Mix C8-C24, Supelco). The peak area was quantified using ChromeCard Software and the results were expressed as mean percentages of total FA.

### 4.4. Total Antioxidant Capacity and Lipid Peroxidation

Antioxidant compounds were extracted by mixing 5 g of yogurt with 15 mL of methanol. The solution was gently shaken for 40 min in the dark and then centrifuged for 15 min at 4000 rpm. Then, the supernatants were filtered and used for analysis. The antioxidant capacity was evaluated through the 2,2-azinobis-3-ethylbenzothiazoline-6-sulfonic acid (ABTS) method according to the protocol described by Chen et al. [42]. Initially, 100 µL of extract was mixed with 1 mL of opportunely diluted ABTS solution, and the colorimetric evaluations were performed at 734 nm after 4 min of incubation at room temperature. An external seven-point calibration curve for Trolox (ranging from 1 to 50 μmol·g^−1^; R^2^ = 0.9961) was prepared for the quantification and the results were reported on a dry matter basis as μmol·g^−1^ Trolox equivalent antioxidant capacity (TEAC).

The TBARs test was used to evaluate the lipid peroxidation through the identification of acid-reactive substances. The analysis was performed in accordance with the procedure previously adopted by Ianni et al. [43], with slight modifications. Five grams of yogurt were mixed with 500 µL of butylated hydroxytoluene (BHT) 0.1% in methanol to stop the oxidation process. At this point, the sample was distilled, and 2 mL of distillate were mixed with an equal volume of thiobarbituric acid (TBA) solution (0.02 M). Finally, the sample was heated for 1 h at 80 °C in a thermostatic bath; after cooling at room temperature, the absorbance at 534 nm was determined for each sample. Data were expressed in µg of malondialdehyde (MDA) equivalent per gram of yogurt.

### 4.5. Determination of Volatile Compounds

The volatile compounds (VOCs) were extracted through a solid-phase microextraction (SPME), and then separated and identificated as previously described [44] with the use of a gas chromatograph (Clarus 580; Perkin Elmer, Walthman, MA, USA) coupled with a mass spectrometry (SQ8S; Perkin Elmer) and equipped with an Elite-5MS column (length: 30 m; internal diameter: 0.25 mm; film thickness: 0.25 μm; Perkin Elmer, Waltham, MA, USA). Ten grams of yogurt were transferred in vials and mixed with 5 mL of NaCl water solution (360 g/L), and 10 µL of 4-methyl-2-heptanone, which was used as internal standard with the aim of evaluating the extraction efficiency downstream of the analysis. After sealing the vial, the sample was stirred at 60 °C in a thermostatic bath, and the adsorption of VOCs was performed with a divinybenzene–carboxen–polydimethylsiloxane SPME fiber exposed for 45 min in the headspace. At this point, the extracted VOCs were thermally desorbed into the GC injector for 1 min in a splitless mode at 250 °C. Helium was used as carrier gas with a flow rate of 1 mL/min and the oven temperature program was started at 50 °C, held for 1 min, then increased up to 200 °C with a ratio of 3 °C/min, then held for 1 min and finally increased up to 250 °C with a ratio of 15 °C/min, then held for 15 min. The mass spectrometer operates in electronic impact ionization mode at 70 eV. Volatile compounds were identified by a comparison with the mass spectra included in the library database (NIST Mass Spectral library (2014), Search Program version 2.0, National Institute of Standards and Technology, US Department of Commerce, Gaithersburg, MD, USA) and by comparing the eluting order with modified Kovats indices, according to the method of Van Den Dool and Kratz [45]. The data were expressed as a percentage of the relative abundance of each compound in relation to the sum of total VOCs.

### 4.6. Statystical Analysis

All the listed evaluations were performed on 12 samples per group (six samples for T1 and six samples for T7), and the analyses on individual samples were performed in triplicate. Results were reported as mean values with corresponding standard deviations (SD). The analysis of statistically significant differences between the two groups of data were performed by using the SigmaPlot 12.0 Software (Systat software, Inc., San Jose, CA, US) for the Windows operating system (ANOVA, Student’s t-test); *p* values lower than 0.05 were considered statistically significant.

## 5. Conclusions

The results shown in this study suggest the positive role of dietary OL supplementation on the nutritional characteristics and volatile profile of goats’ milk yogurt. Dietary OL intake was effective in inducing an increase in the concentration of unsaturated fatty acids, in addition to the general improvement in oxidative stability. This finding could justify an improvement in the shelf-life of the product and was also confirmed by a reduction in the concentration of volatile aldehydes. The characterization of the volatile profile was also useful in highlighting the accumulation of compounds that could justify an improvement in the yogurt flavor, although further sensorial analysis is necessary to evaluate any variations in consumer acceptability.

## Figures and Tables

**Table 1:** Fatty acid composition of yogurt samples obtained from goats fed a standard diet (CG) and goats fed a dietary supplementation of olive leaves (OLG).

Fatty Acids ^1^	CG	OLG	*p*-Value
C4:0	0.54 ± 0.22	0.83 ± 0.39	ns
C6:0	1.03 ± 0.26	1.31 ± 0.39	ns
C8:0	1.73 ± 0.29	1.92 ± 0.34	ns
C10:0	7.65 ± 0.69	7.33 ± 0.58	ns
C12:0	4.31 ± 0.21	3.48 ± 0.12	*
C14:0	11.72 ± 0.44	10.75 ± 0.33	*
C15:0	0.93 ± 0.07	0.92 ± 0.03	ns
C16:0	29.58 ± 0.80	26.67 ± 0.66	*
C17:0	0.67 ± 0.02	0.66 ± 0.01	ns
C18:0	11.04 ± 0.42	13.10 ± 0.74	**
C20:0	0.27 ± 0.03	0.29 ± 0.01	ns
C22:0	0.09 ± 0.03	0.09 ± 0.01	ns
total SFA	69.54 ± 5.80	67.35 ± 5.61	ns
C14:1	0.42 ± 0.02	0.43 ± 0.01	ns
C16:1	0.33 ± 0.01	0.34 ± 0.01	ns
C18:1 *trans*11	0.43 ± 0.01	0.52 ± 0.15	*
C18:1 *cis*9	20.89 ± 0.59	23.22 ± 0.61	**
C18:1 *cis*11	0.38 ± 0.02	0.30 ± 0.01	ns
total MUFA	22.25 ± 0.92	24.80 ± 0.77	*
C18:2	2.92 ± 0.18	2.60 ± 0.14	*
CLA	0.89 ± 0.07	1.12 ± 0.09	*
C18:3	0.78 ± 0.02	1.13 ± 0.05	**
total PUFA	4.62 ± 0.14	4.84 ± 0.07	*
other FAs	3.20 ± 0.31	3.01 ± 0.30	ns
MUFA/SFA	0.32 ± 0.02	0.37 ± 0.03	*
PUFA/SFA	0.07 ± 0.01	0.07 ± 0.02	ns
UFA/SFA	0.39 ± 0.02	0.44 ± 0.04	*
DI C14:1 *cis*-9/(C14:0+C14:1*cis*-9)	0.04 ± 0.01	0.04 ± 0.01	ns
DI C16:1*cis*-9/(C16:0+C16:1*cis*-9)	0.01 ± 0.01	0.01 ± 0.01	ns
DI C18:1*cis*-9/(C18:0+C18:1*cis*-9)	0.65 ± 0.01	0.64 ± 0.01	ns
DI CLA/(C18:1*trans*-11+CLA)	0.62 ± 0.09	0.69 ± 0.02	ns

^1^ Data are reported as mean percentage of total fatty acid methyl esters ± S.D. Saturated fatty acid (SFA); monounsaturated fatty acid (MUFA); polyunsaturated fatty acid (PUFA); conjugated linoleic acids (CLA); desaturation index (DI); not significant (ns); * *p* < 0.05; ** *p* < 0.01.

**Table 2:** Volatile compounds (VOC) identified in yogurt samples obtained from goats fed a standard diet (CG) and goats fed a dietary supplementation of olive leaves (OLG). Samples were analyzed after 1 day (T1) and 7 days of storage (T7) at 4 °C.

VOC ^1^	T1			T7		
	CG	OLG	*p*	CG	OLG	*p*
**FFAs**						
Acetic acid	0.26 ± 0.14	0.40 ± 0.15	ns	nd	0.48 ± 0.20	ns
Butanoic acid	0.27 ± 0.23	0.16 ± 0.06	ns	0.10 ± 0.03	0.11 ± 0.03	ns
Hexanoic acid	18.03 ± 1.69	20.80 ± 2.30	ns	15.39 ± 0.99	13.94 ± 0.74	*
Heptanoic acid	0.56 ± 0.05	0.60 ± 0.06	ns	0.62 ± 0.02	0.43 ± 0.01	**
Octanoic acid	38.91 ± 3.31	45.70 ± 4.28	*	22.99 ± 2.61	43.79 ± 4.46	**
Nonanoic acid	0.29 ± 0.14	0.18 ± 0.03	ns	0.11 ± 0.03	0.14 ± 0.04	ns
Decanoic acid	11.54 ± 0.64	15.11 ± 1.50	*	14.03 ± 2.77	13.97 ± 2.26	ns
**Alcohols**						
1-Hexanol	1.80 ± 0.13	0.88 ± 0.06	**	3.88 ± 0.71	1.72 ± 0.01	*
2-Ethyl-hexan-1-ol	1.76 ± 0.14	1.13 ± 0.11	*	3.41 ± 0.25	2.45 ± 0.19	ns
1-Heptanol	0.95 ± 0.13	0.24 ± 0.03	**	1.06 ± 0.18	0.67 ± 0.09	*
1-Octanol	0.39 ± 0.04	0.17 ± 0.02	**	0.92 ± 0.03	0.39 ± 0.01	**
1-Nonanol	0.81 ± 0.05	0.52 ± 0.02	**	1.32 ± 0.11	0.83 ± 0.10	**
**Aldehydes**						
Heptanal	0.05 ± 0.03	0.02 ± 0.01	ns	0.20 ± 0.10	0.04 ± 0.02	*
Nonanal	1.84 ± 0.17	0.64 ± 0.05	**	4.53 ± 0.25	2.06 ± 0.10	*
2-Heptenal	0.09 ± 0.01	0.08 ± 0.04	ns	0.09 ± 0.01	0.02 ± 0.01	ns
2-Octenal	0.12 ± 0.02	0.07 ± 0.02	ns	0.28 ± 0.01	0.11 ± 0.03	ns
2-Decenal	0.20 ± 0.01	0.22 ± 0.02	ns	0.30 ± 0.04	0.19 ± 0.03	ns
**Esters**						
Butyl heptanoate	0.18 ± 0.01	0.03 ± 0.01	**	0.65 ± 0.05	0.26 ± 0.03	**
**Ketones**						
Acetoin	1.99 ± 0.08	3.20 ± 0.31	**	3.22 ± 1.59	2.10 ± 0.39	ns
2-Pentanone	0.10 ± 0.01	0.04 ± 0.01	ns	0.13 ± 0.02	0.59 ± 0.02	ns
2,3-Pentanedione	0.06 ± 0.02	0.04 ± 0.01	ns	0.67 ± 0.01	0.03 ± 0.05	ns
2-Heptanone	9.30 ± 3.11	4.43 ± 1.02	*	11.32 ± 2.40	6.81 ± 1.11	*
2-Nonanone	9.25 ± 0.74	4.68 ± 0.41	**	12.73 ± 0.99	7.38 ± 0.53	**
2-Undecanone	1.38 ± 0.25	0.98 ± 0.17	ns	1.94 ± 0.46	1.38 ± 0.20	ns
**Lactones**						
δ-Decalactone	0.09 ± 0.01	0.03 ± 0.02	ns	0.05 ± 0.03	0.07 ±0.05	ns
δ-Dodecalactone	0.05 ± 0.01	0.07 ± 0.01	ns	0.07 ± 0.01	0.03 ± 0.01	ns

^1^ Data are reported as mean percentage of total volatile compounds (VOCs) ± S.D; Free fatty acids (FFAs); not detectable (nd); not significant (ns); * *p* < 0.05; ** *p* < 0.01.
